# ECCO_2_R therapy in the ICU: consensus of a European round table meeting

**DOI:** 10.1186/s13054-020-03210-z

**Published:** 2020-08-07

**Authors:** Alain Combes, Georg Auzinger, Gilles Capellier, Damien du Cheyron, Ian Clement, Guglielmo Consales, Wojciech Dabrowski, David De Bels, Francisco Javier González de Molina Ortiz, Antje Gottschalk, Matthias P. Hilty, David Pestaña, Eduardo Sousa, Redmond Tully, Jacques Goldstein, Kai Harenski

**Affiliations:** 1 Sorbonne Université, INSERM, UMRS_1166-ICAN, Institute of Cardiometabolism and Nutrition, 47, Boulevard de l’Hôpital, F-75013 Paris, France; 2 Service de Médecine Intensive-Réanimation, Institut de Cardiologie, APHP Hôpital Pitié–Salpêtrière, F-75013 Paris, France; 3 Department of Critical Care, King’s College Hospital, London, SE5 9RS UK; 4 Department of Critical Care, Cleveland Clinic, London, SW1Y 7AW UK; 5 Service de Médecine Intensive-Réanimation CHRU Besançon, EA 3920 University of Franche Comte, Besançon, France; 6 Australian and New Zealand Intensive Care Research Centre, Department of Epidemiology and Preventive Medicine, Monash University, Melbourne, Australia; 7 Service de Médecine Intensive-Réanimation, Caen University Hospital, 14000 Caen, France; 8 Critical Care Unit, Royal Victoria Infirmary, Newcastle upon Tyne, NE1 4LP UK; 9 Department Emergency and Critical Care, Prato Hospital, Azienda Toscana Centro, Prato, Italy; 10 Department of Anaesthesiology and Intensive Care, Medical University of Lublin, Jaczewskiego Street 8, 20-954 Lublin, Poland; 11 Service des Soins Intensifs Médico-chirurgicaux, CHU Brugmann, 4 Place A Van Gehuchten, 1020 Brussels, Belgium; 12 Department of Critical Care, University Hospital Mútua Terrassa, Universitat de Barcelona, Terrassa, Barcelona Spain; 13 Department of Critical Care, University Hospital Quirón Dexeus, Universitat Autònoma de Barcelona, Barcelona, Spain; 14 Department of Anaesthesiology, Intensive Care Medicine and Pain Medicine, University Hospital Münster, Münster, Germany; 15 Institute of Intensive Care Medicine, University Hospital of Zürich, Rämistrasse 100, 8091 Zürich, Switzerland; 16 Department of Anesthesiology and Surgical Critical Care, Hospital Universitario Ramón y Cajal, IRYCIS, Carretera de Colmenar Viejo km 9, 28034 Madrid, Spain; 17 Universidad de Alcalá de Henares, Madrid, Spain; 18 Serviço de Medicina Intensiva, Centro Hospitalar e Universitário de Coimbra, Praceta Mota Pinto, 3000-075 Coimbra, Portugal; 19 Department of Intensive Care, Royal Oldham Hospital, Northern Care Alliance, Oldham, OL1 2JH UK; 20 Baxter World Trade SPRL, Acute Therapies Global, Braine-l’Alleud, Belgium; 21 Baxter, Baxter Deutschland GmbH, Unterschleissheim, Germany

**Keywords:** Acute respiratory distress syndrome, Chronic obstructive pulmonary disease, CO_2_ removal, Consensus, Driving pressure, ECCO_2_R, Gas exchange, Lung protective ventilation, Tidal volume, Therapy experience

## Abstract

**Background:**

With recent advances in technology, patients with acute respiratory distress syndrome (ARDS) and severe acute exacerbations of chronic obstructive pulmonary disease (ae-COPD) could benefit from extracorporeal CO_2_ removal (ECCO_2_R). However, current evidence in these indications is limited. A European ECCO_2_R Expert Round Table Meeting was convened to further explore the potential for this treatment approach.

**Methods:**

A modified Delphi-based method was used to collate European experts’ views to better understand how ECCO_2_R therapy is applied, identify how patients are selected and how treatment decisions are made, as well as to identify any points of consensus.

**Results:**

Fourteen participants were selected based on known clinical expertise in critical care and in providing respiratory support with ECCO_2_R or extracorporeal membrane oxygenation. ARDS was considered the primary indication for ECCO_2_R therapy (*n* = 7), while 3 participants considered ae-COPD the primary indication. The group agreed that the primary treatment goal of ECCO_2_R therapy in patients with ARDS was to apply ultra-protective lung ventilation via managing CO_2_ levels. Driving pressure (≥ 14 cmH_2_O) followed by plateau pressure (*P*_plat_; ≥ 25 cmH_2_O) was considered the most important criteria for ECCO_2_R initiation. Key treatment targets for patients with ARDS undergoing ECCO_2_R included pH (> 7.30), respiratory rate (< 25 or < 20 breaths/min), driving pressure (< 14 cmH_2_O) and *P*_plat_ (< 25 cmH_2_O). In ae-COPD, there was consensus that, in patients at risk of non-invasive ventilation (NIV) failure, no decrease in PaCO_2_ and no decrease in respiratory rate were key criteria for initiating ECCO_2_R therapy. Key treatment targets in ae-COPD were patient comfort, pH (> 7.30–7.35), respiratory rate (< 20–25 breaths/min), decrease of PaCO_2_ (by 10–20%), weaning from NIV, decrease in HCO_3_^−^ and maintaining haemodynamic stability. Consensus was reached on weaning protocols for both indications. Anticoagulation with intravenous unfractionated heparin was the strategy preferred by the group.

**Conclusions:**

Insights from this group of experienced physicians suggest that ECCO_2_R therapy may be an effective supportive treatment for adults with ARDS or ae-COPD. Further evidence from randomised clinical trials and/or high-quality prospective studies is needed to better guide decision making.

## Background

Advances in technology to deliver extracorporeal carbon dioxide removal (ECCO_2_R) therapy have simplified this approach, making it easier to deploy for the management of adults with both hypoxaemic and hypercapnic acute respiratory failure (ARF) [1–4]. In patients with acute respiratory distress syndrome (ARDS), ECCO_2_R therapy may be used to allow ultra-protective lung ventilation (UPLV) and reduce ventilator-induced lung injury (VILI) by decreasing tidal volume (*V*_T_), both plateau (*P*_plat_) and driving pressures and respiratory rate, while also controlling respiratory acidosis [5–14]. In patients with acute exacerbations of chronic obstructive pulmonary disease (ae-COPD) with severe respiratory acidosis and hypercapnic respiratory failure, ECCO_2_R therapy may be applied to prevent intubation in patients at risk of non-invasive ventilation (NIV) failure [15]. It may also be used to hasten weaning from mechanical ventilation (MV) and early extubation in those who require invasive ventilation [10, 15–17].

However, there is currently limited evidence regarding the use of ECCO_2_R therapy in these indications, with available data limited to the description of single cases or to case series that include a small number of patients [16, 18–21], as well as a few retrospective matched cohort studies [15, 22]. Additionally, questions remain on how best to implement a therapy that might be associated with serious side-effects [1]. Ongoing and published trials such as VENT-AVOID (NCT03255057), REST (NCT02654327) [2] and SUPERNOVA (NCT02282657) [11, 12, 23] are expected to provide valuable evidence to support decision making.

Given the potential of ECCO_2_R therapy to provide effective supportive treatment for a wide range of patient groups, we convened a European ECCO_2_R therapy Expert Round Table Meeting to better understand how ECCO_2_R therapy is applied in key diagnostic groups, e.g. patients with ARDS or ae-COPD, identify how patients are selected, understand how treatment decisions are made and delineate areas of consensus in the group.

## Methods

### Research questions and objectives

The ECCO_2_R therapy Expert Round Table Meeting was held in Brussels in July 2019 and was attended by 14 clinicians who regularly provide ECCO_2_R therapy in hospitals across Europe in order to provide a European perspective on ECCO_2_R therapy. Each attendee was a senior clinician/intensivist invited based on their experience delivering ECCO_2_R therapy, with and without continuous renal replacement therapy, using different devices. The attendees had direct clinical experience with a wide range of ECCO_2_R devices, including ALung, iLA, Prismalung and PALP (the later had been removed from the market at the time of the meeting due to loss of the distribution agreement). In addition, several of the attendees are principal investigators in recently completed or ongoing clinical trials, including randomised controlled trials such as REST and SUPERNOVA. Conflict of interest declarations for the attendees can be found at the end of the manuscript.

The meeting objectives were to better define and understand the application of ECCO_2_R therapy in key indications (ARDS and ae-COPD), to identify patient selection criteria and when to initiate and stop/wean patients from treatment and to determine points of consensus and differences in clinical practice in those centres represented at the meeting. A non-systematic search of MEDLINE, ClinicalTrials.gov and other sites was performed to identify key studies and trials to support the development of the questions and the content of the meeting.

### Data collection and analysis

**Fig. 1:**
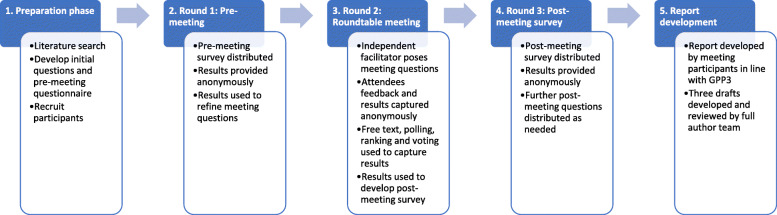
Overview of the five-step Delphi method used in the Round Table Meeting. Each step was a distinct process that was completed before the following step was initiated. Results and discussions from each step were independently analysed and used to inform the direction and content of the following steps, e.g. if the group were split on a topic, then clarifying questions were crafted to guide the discussions in the following step(s) to identify and explore points of consensus or difference. GPP3, Good Publication Practice 3

A modified Delphi-based method (Fig. 1) was used to collate the clinicians’ views in three rounds of questioning [24]. The meeting questions as well as the pre-meeting and post-meeting questionnaires were developed by JG and KH before being reviewed and approved by AC. JG and KH were present as Baxter employees and moderators, but were not permitted to provide answers or responses, either to the survey questions or during the meeting. Round 1 data were collected via an interactive PDF questionnaire circulated in advance of the meeting, and results were analysed anonymously. Round 2 data were collected during the meeting, attendees were divided into 4 subgroups and the questions were presented by an independent facilitator. Open questions were used to encourage freedom of response, and the meeting was designed to allow the attendees adequate time to consider and respond to the questions based on their experience. Attendees could respond to the questions either through anonymous electronic voting or by inputting responses into a microcomputer, with responses collected and discussed openly by the group. Round 3 was a second interactive PDF questionnaire, circulated post-meeting, designed to follow up on discussion points raised at the face-to-face meeting, with results analysed anonymously. Details on the process for information gathering and the questions are provided in Additional file 1.

Target values for ventilation parameters of interest—criteria for initiation of ECCO_2_R therapy and treatment targets for ECCO_2_R therapy in both ARDS and ae-COPD—were collected during the three rounds of questioning. These values were subsequently evaluated for consensus. To facilitate the analysis of the responses for certain questions, a scoring system was employed. Participants were asked to score their responses in order of importance, giving them a score (e.g. from 1 to 8, depending on the number of variables). Scores were then combined to give a total score for each parameter, with higher scores indicating a higher perceived importance. To determine whether a consensus was reached or not based on participant responses to the questions, a threshold of ≥ 80% of participants in agreement was used to define if consensus was reached, a level that has been used in previous analyses [25]. Majority agreement indicates that ≥ 50% of participants agreed, but consensus level was not reached, and no agreement means that < 50% of participants agreed. The report was drafted by an independent medical writing company (SciMentum, Nucleus Global) and paid for by Baxter in line with Good Publication Practice 3. The various drafts were reviewed and approved by AC before being reviewed by the full author team. All authors provided their approval to submit and meet the ICMJE criteria for authorship.

## Results

### Attendee clinical experience

Twelve clinicians completed the pre-meeting survey: eight worked in Combined Surgical and Medical intensive care units (ICUs), while the others were employed in Medical ICUs (*n* = 2), Surgical ICUs (*n* = 2) and Cardiac Surgery ICUs (*n* = 2); respondents could be employed at more than one type of centre. ICUs had a median of 20 beds/unit and 400–2000 admissions/year. Extracorporeal membrane oxygenation (ECMO) experience of participants ranged from 0 to 80 veno-venous ECMO procedures/year and 0 to 220 veno-arterial ECMO procedures/year.

### Indications and rationale for ECCO_2_R based on pre-meeting survey

Analysis of the Round 1 pre-meeting survey responses revealed that ARDS was considered the primary indication for ECCO_2_R therapy by 7 participants, while 3 participants considered ae-COPD to be the primary indication. Severe asthma was also mentioned as another potential ECCO_2_R indication, although less frequently. The median number of ARDS admissions (as per the Berlin definition [26]) was 60 patients per centre per year, with some centres admitting up to 500 patients per year. While the most common criteria stated in the pre-meeting responses for initiating ECCO_2_R therapy in patients with ARDS were to manage hypercapnia with acidosis, although specific criteria varied across the ICUs, likewise, weaning criteria shared at Round 1 varied significantly, with no clearly consistent management pattern being identified between centres. However, most participants (92%) indicated that they would place patients with ARDS in the prone position when using ECCO_2_R therapy. The number of ae-COPD admissions ranged from 0 to 250 patients per centre per year (median 50). Participants indicated that ECCO_2_R therapy was predominantly initiated to prevent intubation in patients at risk of NIV failure or to facilitate extubation in patients who had been intubated after NIV failure.

### Use of ECCO_2_R therapy in patients with ARDS

During the Expert Round Table Meeting and post-meeting survey (Rounds 2 and 3, respectively), the group considered the ventilation parameters for implementation of a lung protective ventilation (LPV) strategy in all patients with ARDS and agreed upon the following targets: driving pressure, 10–14 cmH_2_O; positive end-expiratory pressure (PEEP), 10–14 cmH_2_O; *P*_plat_, 25–29 cmH_2_O; and respiratory rate either 20–25 or 25–30 breaths/min, although most of the group would target a respiratory rate of 25 breaths/min. There was some variation in responses among the group when asked about target pH, with half of participants opting for a target pH value of 7.25–7.30, while others indicated the target should be > 7.30 (*n* = 4), < 7.25–7.30 (*n* = 2) or < 7.20 (*n* = 1). Finally, the panellists thought *V*_T_ should be set at 6.0 mL/kg of predicted body weight (PBW), although 6.1–7.0 or 7.1–8.0 mL/kg PBW were also considered to be reasonable targets. When asked in the post-meeting survey (Round 3) about the preferred ventilation mode used for patients with ARDS undergoing LPV, the group were split with respect to pressure control (pressure assist) (*n* = 8) and flow control (volume assist) (*n* = 6) modes of ventilation. These recommendations agreed with the most recent guidelines for the ventilation management of patients with ARDS [27, 28].

There was consensus among the group (91% [2 participants were unavailable for this question, 11 of *n* = 12/14 voted in favour]) that the primary treatment goal of ECCO_2_R therapy for patients with ARDS was to apply UPLV via managing CO_2_ levels. For initiating ECCO_2_R therapy in patients with ARDS, driving pressure (≥ 14 cmH_2_O) followed by *P*_plat_ (≥ 25 cmH_2_O) was considered the most important criteria, and this was confirmed in the post-meeting survey (Tables 1 and 2). Additional key parameters included pH (< 7.25), reducing *V*_T_ to < 6 mL/kg PBW, PaCO_2_ (> 60–80 mmHg), respiratory rate (≥ 25 to > 30 breaths/min), PaO_2_/FiO_2_ (100–200) and PEEP (combined findings from Rounds 2 and 3).

**Table 1:** ECCO_2_R treatment criteria for patients with ARDS

Parameter	Target	Score	
**Initiation criteria**			
Driving pressure	≥ 14 cmH_2_O	31	Consensus
*P*_plat_	≥ 25 cmH_2_O	22	Consensus
PaCO_2_	> 60–80 mmHg	21	Majority agreement
pH	< 7.25	20	Majority agreement
Reduce V_T_ to < 6 mL/PBW	–	18	Majority agreement
Respiratory rate	≥ 25 to > 30	14	Majority agreement
PaO_2_/FiO_2_	100–200	10	Majority agreement
PEEP	–	8	No agreement
**Treatment targets**			
Driving pressure	< 14 cmH_2_O	66*	Consensus
*P*_plat_	< 25 cmH_2_O	57*	Majority agreement^†^
Respiratory rate	< 25 or < 20 breaths/min	44*	Consensus
pH	> 7.30	39*	Majority agreement
*V*_T_	≤6 mL/PBW	39*	Majority agreement
PaCO_2_	< 50–55 mmHg	30	Majority agreement

Criteria for ECCO_2_R treatment considered to be of importance and selected from the provided list. Target describes any potential target values identified, with ‘–’ indicating that no target parameter was provided or considered relevant. Score indicates the combined total score, with higher scores indicating a higher perceived importance. Consensus means a consensus threshold (≥ 80%) was reached, majority agreement means ≥ 50% agreed but consensus level was not reached, and no agreement means < 50% agreed

*Based on the post-meeting survey. ^†^Note, for *P*_plat_, a consensus threshold of 80% was not reached in the meeting; in the post-meeting survey, it was rated as the second most important target

**Table 2:** Typical characteristics for initiating ECCO_2_R for rescue therapy and to facilitate ultra-protective ventilation in ARDS

Parameter	Target for initiation in: Rescue	Target for initiation in: Ultra-protective ventilation
Driving pressure	> 15 to 20 cmH_2_O	> 13 to 15 cmH_2_O
*P*_plat_	> 30 to 35 cmH_2_O	≥ 25 cmH_2_O
PaCO_2_	≥ 60 mmHg	≥ 60 mmHg
pH	< 7.25–7.30	< 7.25–7.30
Respiratory rate	> 20 to 30 breaths/min	> 20 breaths/min
PaO_2_/FiO_2_	< 150	< 150
PEEP	> 8 to 15	≥ 8

Responses were captured during the post-meeting survey (Round 3) and general themes were identified

Participants were evenly split during the meeting on the primary rationale for ECCO_2_R therapy, being rescue therapy in patients with ARDS undergoing injurious MV, i.e. those with very high plateau and driving pressures despite reduced *V*_T_ and PEEP (*n* = 7), or to facilitate UPLV to prevent the deleterious effects of MV in patients already undergoing LPV (*n* = 7). Based on the results of the post-meeting survey, a consensus was reached among the group (12/14, 86% of participants) that ECCO_2_R was a strategy they would consider selecting for rescue in patients with ARDS. Typical characteristics for initiating ECCO_2_R in a rescue situation obtained as part of the post-meeting survey are summarised in Table 2. A majority (10/14, 71% of participants) indicated that they would select ECCO_2_R as a means of facilitating UPLV for patients with ARDS, and typical characteristics for selecting patients are summarised in Table 2.

For both potential indications, patients would not be considered suitable for an ECCO_2_R strategy if they met the indications for ECMO, such as severe or refractory ARDS [29] and presence of severe right heart failure (ECMO may be a more adequate treatment for these patients), in cases where anticoagulation is contraindicated and for those with major comorbidities and/or predicted survival of < 1 year.

The group considered treatment targets for their patients with ARDS undergoing ECCO_2_R. A consensus was reached regarding driving pressure (< 14 cmH_2_O) and respiratory rate (< 25 or < 20 breaths/min). There was majority agreement with respect to targets for *P*_plat_ (< 25 cmH_2_O), pH (> 7.30 [Rounds 2 and 3]), PaCO_2_ (< 50 or < 55 mmHg) and *V*_T_ (≤ 6 mL/kg PBW). Other target parameters were not proposed by the group (Table 1). The expected average length of time patients with ARDS would remain on ECCO_2_R therapy was suggested to be 1–3 days (*n* = 5) and 4–6 days (*n* = 9).

Following discussion during the meeting on a protocol for weaning from ECCO_2_R in patients with ARDS, a protocol was proposed and reviewed as part of the post-meeting survey (Table 3). The group voted on each step and reached consensus (92% of participants, *n* = 13) that this proposal was a suitable weaning strategy.

**Table 3:** ECCO_2_R weaning protocol for patients with ARDS

Weaning criteria and steps for weaning for ECCO_**2**_R in ARDS*	
ECCO_2_R will be applied for at least 48 h	
PaO_2_/FiO_2_ > 200 mmHg before testing weaning possibility	
Set *V*_T_ at 6 mL/PBW and PEEP 5–10 cmH_2_O	
Driving pressure should be < 14 cmH_2_O	
Respiratory rate should be 20–30 breaths/min	
Reduce gas flow to zero, using 2 L/min decremental steps	
While weaning, pH should remain > 7.30 and respiratory rate < 25 breaths/min	
Patient will be weaned off ECCO_2_R therapy after a minimum of 12 h of stability under these settings (including pH > 7.30 and respiratory rate < 25 breaths/min)	

*A consensus was reached for all of these criteria and steps

### Use of ECCO_2_R therapy in patients with ae-COPD

There was consensus during the meeting that patients with ae-COPD who should receive ECCO_2_R therapy were those at risk of NIV failure, as well as patients recently initiated on MV after NIV failure to allow for early extubation within 24 h of initiating ECCO_2_R therapy. Other patient groups would be considered (e.g. patients on prolonged MV who require weaning from invasive ventilation and patients who are refusing intubation), but a consensus was not reached.

The group agreed that for patients with ae-COPD at risk of NIV failure, ‘no decrease in PaCO_2_’ and ‘no decrease in respiratory rate’ while on NIV were both key initiation criteria for ECCO_2_R therapy (Table 4). These criteria were considered indicative of NIV failure. Clinical signs of respiratory failure and pH (< 7.25 [*n* = 5] or 7.25–7.30 [*n* = 6]) would be considered as initiation criteria by most of the participants. Baseline PaCO_2_ and respiratory rate as main triggers were favoured by less than half of participants. For patients with ae-COPD who had already been intubated, criteria for initiating ECCO_2_R therapy varied (Table 4).

**Table 4:** ECCO_2_R treatment initiation criteria for patients with ae-COPD

**Initiation criteria for patients at risk of NIV failure**	
**Parameter**	
No decrease in PaCO_2_ while on NIV	Consensus
No decrease in respiratory rate while on NIV	Consensus
Clinical signs of respiratory failure	Majority agreement
pH 7.25–7.30	Majority agreement
Baseline PaCO_2_	No agreement
Baseline respiratory rate	No agreement
**Initiation criteria for patients who are already intubated**	
- Patients who look like they will not be extubated early without ECCO_2_R	
○ Previous intubation for ae-COPD	
○ Has failed a spontaneous breathing trial due to increased dyspnoea	
○ Reintubation after first extubation attempt despite NIV	
○ Patients with severe bronchospasm who are difficult/impossible to ventilate adequately or otherwise not responding to medical treatment	
○ Patients who remain hypercapnic and not improving with MV	
- No hypoxemia preventing extubation	
- MV < 72 h	
- Patients with home NIV and good quality of life	

Criteria for ECCO_2_R treatment considered to be of importance and selected from the provided list. Target describes any potential target values identified. Consensus means a consensus threshold (≥ 80%) was reached, majority agreement means ≥ 50% agreed but consensus level was not reached, and no agreement means < 50% agreed

Scoring and ranking was not conducted for this section during the meeting

Factors for excluding patients with ae-COPD from ECCO_2_R typically included patients with end-stage disease (the group highlighted that markers for this include severe functional limitation and cachexia); contraindications to anticoagulation; problems with vascular access; patient’s wishes, e.g. refusal to be intubated, except in cases where ECCO_2_R therapy represented the last resource accepted by the patient; poor quality of life; and the patient not being a candidate for MV.

Treatment targets for patients with ae-COPD receiving ECCO_2_R therapy were, in order of perceived importance (Table 5), comfortable patient, pH (> 7.35/7.30; no consensus on specific pH), respiratory rate (< 20–25 breaths/min), decrease of PaCO_2_ by 10–20%, weaning from NIV, decrease in HCO_3_^−^ and maintaining haemodynamic stability. Consensus on a weaning protocol for patients with ae-COPD was reached during the meeting (Table 5).

**Table 5:** ECCO_2_R treatment targets and weaning protocol for patients with ae-COPD

**Treatment targets for patients with ae-COPD**		
**Parameter**	**Target**	**Score**
Comfortable patient	–	27
pH	> 7.35/7.30, no consensus on specific pH	23
Respiratory rate	< 20–25 breaths/min	19
Decrease of PaCO_2_ by 10–20%	–	18
Weaning from NIV	–	9
Decrease in HCO_3_^−^	–	9
Maintaining haemodynamic stability	–	7
**ECCO**_**2**_**R weaning protocol for patients with ae-COPD**		
1. Patient weaned from NIV for > 6 h		
a. Excluding patients on home NIV or candidates for long-term NIV		
2. Intubated patients weaned from MV for > 6 h		
3. SpO_2_ ≥ 88% with supplemental O_2_ if needed		
4. Reduce sweep gas flow rate by 1–3 L/min; check arterial blood gas after 1 h for:		
a. pH ≥7.35 with respiratory rate < 25 breaths/min		
b. PaO_2_ > 55 mmHg		
c. SpO_2_ > 88%		
d. FiO_2_ < 40%		
5. Repeat sweep gas reduction until zero gas flow reached, while arterial blood gas targets maintained		
6. Remove ECCO_2_R after 6 h of stability of the aforementioned criteria		

Treatment targets for ECCO_2_R considered to be of importance and selected from the provided list. Target describes any potential target values identified. Score indicates the combined total score, with higher scores indicating a higher perceived importance. Consensus means a consensus threshold (≥ 80%) was reached, majority agreement means ≥ 50% agreed but consensus level was not reached, and no agreement means < 50% agreed. The ECCO_2_R weaning protocol for patients with ae-COPD was developed and voted on during the meeting, with all attendees in agreement

### Anticoagulation strategy for patients receiving ECCO_2_R

Responses obtained during Round 1 (pre-meeting survey) showed that heparin was the preferred choice of anticoagulant used during ECCO_2_R therapy (~ 80% of participants stated that heparin was their anticoagulant of choice). This was confirmed in the post-meeting survey, in which unfractionated heparin was the anticoagulant of choice for the majority (~ 90% of participants). The proposed heparin anticoagulation protocol agreed by the group is shown in Table 6. Lastly, argatroban was the group’s preferred anticoagulant in case of proven heparin-induced thrombocytopenia (HIT).

**Table 6:** Heparin anticoagulation strategy

1. Anticoagulation with intravenous unfractionated heparin, preferably applied to the extracorporeal circuit	
2. Monitor aPTT or anti-Xa or both	
a. To obtain an aPTT of 1.5–2.0 times normal baseline (45–70 s), or anti-Xa activity of 0.3–0.5 UI/mL	
3. Initial bolus of heparin	
a. 40–80 units/kg PBW	
b. Bolus will not be performed in patients already on full anticoagulation	
c. Bolus routinely performed when guidewires have been inserted/or after catheter insertion	
4. Patients with proven HIT-2	
a. Argatroban protocol, e.g. 0.5–2.0 μg/kg/min	

## Discussion

The responses obtained from the Expert Round Table Meeting and accompanying pre- and post-meeting surveys have provided further insights into the use of ECCO_2_R therapy across Europe. During a typical Delphi process [24], 100% agreement is rare, and any consensus is the result of multiple rounds of voting and discussion that lead to a convergence of opinion. However, in areas where clinical evidence is limited, as is the case for ECCO_2_R therapy in patients with ARDS and ae-COPD, using a modified Delphi method may offer insight into the current practice of experienced users, which could help inform decision making in local clinical practice. Additionally, the use of the Delphi method to guide these discussions and reach points of consensus will be of potential benefit for the design of future trials. Specifically, the discussions provide insight relevant to inclusion criteria, guidance on the management of patients while receiving ECCO_2_R therapy and possible primary and secondary endpoints.

Key areas of consensus for the use of ECCO_2_R therapy in the treatment of patients with ARDS or ae-COPD were identified. There was consensus among the group that the primary treatment goal of ECCO_2_R therapy for patients with ARDS was to apply UPLV via managing CO_2_ levels; this is in agreement with the findings of a systematic literature review [30]. The group reached a consensus that, when initiating ECCO_2_R therapy in patients with ARDS, driving pressure (≥ 14 cmH_2_O) followed by *P*_plat_ (≥ 25 cmH_2_O) was the most important criteria to consider. Higher PEEP, lower peak and plateau pressures and lower respiratory rate have been shown to correlate with improved survival in patients with ARDS [7, 11, 31]. However, only the driving pressure was associated with increased mortality using a multilevel mediation analysis in a large retrospective cohort study of patients with ARDS [32]. It is therefore perhaps not surprising that the key treatment targets for ECCO_2_R in ARDS identified by the group were reductions in driving pressure and respiratory rate.

A pH of < 7.25 was also considered by most of the group to be a criterion for initiation of ECCO_2_R therapy in this patient group. Indeed, a lower pH was recently shown to be independently associated with ICU mortality in the large prospective LUNG SAFE registry [31]. Most of the group also agreed that ECCO_2_R should be initiated at PaCO_2_ levels > 60–80 mmHg. While it was suggested that permissive hypercapnia provided protection against lung injury in terms of lung permeability, oxygenation and lung mechanics [33], more recent data have shown a positive correlation between hypercapnic acidosis and mortality [34, 35]. Raising pH (> 7.30 or > 7.25) and decreasing PaCO_2_ levels were considered important treatment targets, indicating that there is a perception that ECCO_2_R is an important therapy for the management of respiratory acidosis.

The experts were evenly split on the primary rationale for ECCO_2_R therapy, either as a rescue therapy in patients with ARDS undergoing injurious MV, or to facilitate UPLV to prevent VILI. The results from the post-meeting survey highlighted that the group agreed that they would at least consider selecting ECCO_2_R as a strategy in both settings. Ongoing (NCT02654327) [11] randomised trials may help clarify the role of ECCO_2_R, allowing UPLV in patients with acute hypoxemic respiratory failure.

To the best of our knowledge, this is the first publication of a proposed weaning strategy for ECCO_2_R in patients with ARDS. The group reached a consensus regarding a strategy for weaning patients from ECCO_2_R in this setting. It was agreed that ECCO_2_R therapy should be applied for at least 48 h in patients with ARDS, and that a test for PaO_2_/FiO_2_ > 200 mmHg while maintaining a driving pressure < 14 cmH_2_O should be carried out to determine weaning possibility. It was also agreed that patients should be stable for a minimum of 12 h at the ventilation parameters outlined (see Table 3) before any weaning attempt takes place [11].

In a randomised study exploring the role of helium/oxygen in ae-COPD, the rate of patients failing on NIV and requiring MV was 15% [36]. Identifying the subgroup of patients with ae-COPD at high risk of NIV failure is indeed crucial to improve their outcomes by deploying effective preventive strategies. The panel identified ‘lack of decrease in PaCO_2_’ and ‘respiratory rate during NIV’ as important indicators of increased risk of NIV failure and an indication for ECCO_2_R initiation. The group also felt that it was important to allow enough time to show that NIV was ineffective before initiating ECCO_2_R therapy. Furthermore, there are numerous factors involved in NIV failure, and the benefit of ECCO_2_R for this patient group is still a matter of debate due to lack of data from randomised clinical trials [15, 22].

For patients with ae-COPD who are already intubated, the intended use of ECCO_2_R therapy is to rapidly allow extubation, to facilitate oral nutrition and early physiotherapy and to prevent muscle deconditioning [3]. Treatment targets identified by the group clearly fit in with the strategy of reducing the duration of MV and are in line with published data and wider views on the use of ECCO_2_R therapy [1, 19]. The VENT-AVOID trial (NCT03255057) is currently randomising patients to further investigate the benefits of ECCO_2_R therapy in patients at risk of NIV failure or who already have been intubated after NIV failure.

Anticoagulation with intravenous unfractionated heparin was the preferred strategy of the group. This reflects recent studies in the literature in which unfractionated heparin appears to be the anticoagulant most frequently used in this setting [10, 11]. The post-meeting survey highlighted that anticoagulant activity should be monitored using activated partial thromboplastin time (aPTT) and/or anti-Xa; the monitoring approach remains dependent on local practice. For patients with proven HIT, argatroban was the group’s preferred anticoagulant [37, 38].

### Limitations

The findings presented here relate to the experiences of a relatively small number of physicians from centres across Europe; evidence from a larger group of intensivists from multiple regions of the world may be required to support these observations. Certain topics were not covered due to the scope of the meeting. Firstly, the questions covered current practice and did not explore if practices, e.g. inclusion policies of the respective centres, had changed over time. Secondly, certain rarer indications, e.g. lung transplant, were not covered, as the meeting focussed on the broader population of patients requiring ECCO_2_R therapy, e.g. patients with ARDS or ae-COPD. These questions could be covered as part of a follow-up meeting. Additionally, while the authors took every opportunity to ensure all relevant major articles were cited, the purpose of the meeting was to understand current practice as opposed to conducting a comprehensive literature analysis. Finally, the experiences outlined are the physicians’ respective personal experiences and are not a replacement for formal guidelines. The reader should consider their patients’ needs and local guidelines when performing ECCO_2_R therapy.

## Conclusions

The insights from this group of experienced physicians suggested that ECCO_2_R therapy may be a useful and effective supportive treatment for adults in the ICU with both ARDS and ae-COPD. They have however highlighted an urgent need for further evidence in the form of randomised clinical trials and/or high-quality prospective studies to help guide decision making. Ongoing and published trials such as VENT-AVOID (NCT03255057), REST (NCT02654327) [2] and SUPERNOVA (NCT02282657) [11, 12, 23] should provide the data to support these guidelines.

## Supplementary information

**Additional file 1: Expanded methods.** Details on the process for information gathering and the questions.

## Data Availability

Not applicable.

## Abbreviations

ae-COPD: Acute exacerbations of chronic obstructive pulmonary disease
aPTT: Activated partial thromboplastin time
ARDS: Acute respiratory distress syndrome
ARF: Acute respiratory failure
ECCO_2_R: Extracorporeal carbon dioxide removal
ECMO: Extracorporeal membrane oxygenation
FiO_2_: Fraction of inspired oxygen
HIT: Heparin-induced thrombocytopenia
ICU: Intensive care unit
LPV: Lung protective ventilation
MV: Mechanical ventilation
NIV: Non-invasive ventilation
PaCO_2_: Partial pressure of carbon dioxide
PBW: Predicted body weight
PEEP: Positive end-expiratory pressure
*P*_plat_: Plateau pressure
SpO_2_: Oxygen saturation
UPLV: Ultra-protective lung ventilation
VILI: Ventilator-induced lung injury
*V*_T_: Tidal volume
