# Supplementary material for: ECCO2R therapy in the ICU: consensus of a European round table meeting
Source: Crit Care. 2020 Aug 7;24:490. doi: 10.1186/s13054-020-03210-z (PMC7412288; doi:10.1186/s13054-020-03210-z)
Supplement: Supplementary file 1 — Additional file 1: Expanded methods. Details on the process for information gathering and the questions. [file 13054_2020_3210_MOESM1_ESM.docx]

**ECCO_2_R therapy in the ICU: consensus of a European round table meeting**

**ADDITIONAL INFORMATION**

**Expanded methods**

A panel of European experts was selected based on known clinical expertise in the areas of critical care and the use of extracorporeal carbon dioxide removal (ECCO_2_R) therapy in their clinical practice. Feedback on the application of ECCO_2_R therapy in key indications (acute respiratory distress syndrome [ARDS] and acute exacerbations of chronic obstructive pulmonary disease [ae-COPD]) was obtained using a multi-stage iterative blinded process that was adapted from the Delphi method; for further details please refer to the work by Dalkey and Helmer. Briefly, participants were exposed to three rounds of questions to identify patient selection criteria, when to initiate treatment, when to stop/wean patients from treatment for each indication and to determine points of consensus and differences in clinical practice from the represented centres. Questionnaire responses were provided as anonymous free text to avoid identifying the responder and voting was blinded. The three rounds are described in more detail below.

**Round 1**

Before the face-to-face meeting, each participant was sent an interactive PDF questionnaire designed to gain anonymous insights into the use of ECCO_2_R in both ARDS and ae-COPD (see below). Answers were anonymised by a third party before being collated. Participants were asked about the type of intensive care unit (ICU) they work at, its size (number of beds) and frequency of use of ECCO_2_R and extracorporeal membrane oxygenation (ECMO). Information on the primary indication for the use of the ECCO_2_R therapy, rationale for its use, the preferred anticoagulation strategy and devices used was collected.

**Round 2**

The second round was conducted during a face-to-face meeting. Participants were randomly divided into four subgroups and an independent facilitator posed questions that had been developed based on the responses to the Round 1 survey. Responses were obtained using either free text input via a computer terminal following free discussions or blinded voting. The criteria identified for initiation of ECCO_2_R therapy in key indications were ranked by the four groups to gain a consensus overview of their importance, as denoted by an aggregated score; this process was repeated to determine key treatment targets.

**Round 3**

Following the meeting, each participant was sent another interactive PDF questionnaire designed to gain additional insights into the issues discussed during the Round 2 meeting including further exploration of the rationale of using ECCO_2_R therapy, the relative of importance of different criteria identified for initiating ECCO_2_R, the optimal weaning protocol for ECCO_2_R in ARDS patients and to determine whether the discussions at the meeting had impacted clinical practice (see below).

**Pre-meeting questions (Round 1)**

1. What type of unit do you work at? (Please check all that apply.)
   1. Medical ICU
   2. Surgical ICU
   3. Combined medical and surgical ICU
   4. Cardiac surgery ICU
   5. Respiratory acute care
   6. Other (please specify):
2. How many beds are there in your ICU/unit?
3. How many admissions do you have to your ICU/unit per year?
4. What is the average number of patients treated with ECCO_2_R in your ICU/unit per year?
5. What is the average number of patients treated with ECMO in your ICU/unit per year?
   1. VV ECMO: patients/year
   2. VA ECMO: patients/year
6. What are the three primary indications for ECCO_2_R therapy in your ICU/unit? (Please rank the clinical syndromes by importance from i to iii.)
7. ARDS patients in your ICU/unit:
   1. What is the average number of ARDS patients admitted per year?
   2. What is the average number of ARDS patients treated with ECCO_2_R? (If 0, please proceed to Question 8.)
   3. What are your criteria for initiating ECCO_2_R in an ARDS patient?
   4. Do you place the patient in the prone position for ECCO_2_R therapy? (Yes/No)
   5. What are your criteria to wean a patient from ECCO_2_R?
8. Acute exacerbated COPD (ae-COPD) in your ICU/unit:
   1. What is the average number of ae-COPD patients admitted per year?
   2. What is the average number of ae-COPD patients treated with ECCO_2_R? (If 0, please proceed to Question 9.)
   3. Why would you consider ECCO_2_R for ae-COPD patients?
   4. What are your criteria for initiating ECCO_2_R in a patient with ae-COPD?
   5. What are your criteria to wean a patient from ECCO_2_R?
9. What is your preferred anticoagulation option for ECCO_2_R?
10. Which ECCO_2_R devices do you use? (Please list the product name(s))

**Post-meeting questions (Round 3)**

1. Please specify the typical pH value you target for patients with ARDS undergoing lung protective ventilation
   1. >7.30
   2. 7.25–7.30
   3. <7.20–7.25
   4. <7.20
2. Which ventilation mode do you primarily use for patients with ARDS undergoing lung protective ventilation?
   1. Pressure assist control
   2. Volume assist control
3. Would you select ECCO_2_R as a rescue therapy for patients with ARDS? (Yes/No)
   1. If YES, please describe the typical characteristics and, if applicable, the targets you would use for initiating ECCO_2_R as a rescue therapy for patients with ARDS, e.g. driving pressure, plateau pressure (P_plat_), respiratory rate (RR), positive end expiratory pressure (PEEP), tidal volume (V_T_), pH, other?
   2. Are there patients for whom you would not consider using this strategy?
4. Would you select ECCO_2_R as a therapy to allow ultraprotective ventilation for patients with ARDS? (Yes/No)
   1. If YES, please describe the typical characteristics and, if applicable, the targets you would use for initiating ECCO_2_R as a rescue therapy for patients with ARDS, e.g. driving pressure, P_plat_, RR, PEEP, V_T_, pH, other?
   2. Are there patients for whom you would not consider using this strategy?
5. If you considered both strategies applicable, please indicate what percent of patients they would represent (rescue [%], therapy to allow ultraprotective ventilation [%]).
6. When considering the relative importance of criteria for initiating ECCO_2_R therapy in patients with ARDS, driving pressure was ranked as the most important; however, the relative importance of the other criteria was unclear from the meeting. Please rank the importance of the following criteria, with 1 being the most important and 4 the least important (P_plat_, partial pressure of carbon dioxide (PaCO_2_), pH, reduction of V_T_ to <6 mL/kg to allow ultraprotective ventilation).
7. Please indicate the pH you would target when using ECCO_2_R therapy for patients with ARDS.
   1. >7.30
   2. 7.25–7.30
   3. <7.25
8. Please indicate the P_plat_ (cmH_2_O) you would target when using ECCO_2_R therapy for patients with ARDS.
   1. <30
   2. <25
   3. <20
9. Please indicate the pH for initiating ECCO_2_R therapy in patients with ae-COPD.
   1. >7.30
   2. 7.25–7.30
   3. <7.25
   4. I do not use pH
10. For patients with ae-COPD at risk of non-invasive ventilation (NIV) failure, how long do you wait to confirm the ECCO_2_R therapy is working?
11. Proposed protocol for weaning patients with ARDS:
    - ECCO_2_R will be applied for at least 48 hours
    - PaO_2_/fraction of inspired oxygen (FiO_2_) > 200 mmHg for testing weanability
    - Set V_T_ at 6 mL/per body weight (PBW) and PEEP 5–10 cmH_2_O
    - ΔP should be <14 cmH_2_O
    - RR should be 20–30/min
    - Reduce gas flow to zero, using 2 L/min decremental steps
    - pH should remain >7.30 and RR < 25/min
    - Patient will be weaned off ECCO_2_R therapy after a minimum of 12 hours of stability under these settings (including pH > 7.30 and RR < 25/min)
12. Do you agree with Point 1 of the protocol – ‘ECCO_2_R will be applied for at least 48 hours’? (Yes/No – If No, please provide details).
13. Do you agree with Point 2 of the protocol – ‘PaO_2_/FiO_2_ > 200 mmHg for testing weanability’? (Yes/No – If No, please provide details).
14. Do you agree with Point 3 of the protocol – ‘Set V_T_ at 6 mL/PBW and PEEP 5–10 cmH_2_O’? (Yes/No – If No, please provide details).
15. Do you agree with Point 4 of the protocol – ‘ΔP should be <14 cmH_2_O’? (Yes/No – If No, please provide details).
16. Do you agree with Point 5 of the protocol – ‘RR should be 20–30/min’? (Yes/No – If No, please provide details).
17. Do you agree with Point 6 of the protocol – ‘Reduce gas flow to zero, using 2 L/min decremental steps’? (Yes/No – If No, please provide details).
18. Do you agree with Point 7 of the protocol – ‘pH should remain >7.30 and RR < 25/min’? (Yes/No – If No, please provide details).
19. Do you agree with Point 8 of the protocol – ‘Patient will be weaned off ECCO_2_R therapy after a minimum of 12 hours of stability under these settings (including pH > 7.30 and RR < 25/min)’? (Yes/No – If No, please provide details).
20. Have you changed your clinical practice/prescription behaviour related to ECCO_2_R therapy after participating in the ECCO_2_R Expert User Group meeting? (Yes/No – If No, please provide details).
21. From your point of view, what topics require further discussion?
22. Anticoagulation on ECCO_2_R therapy.
    1. Which drug would you use as first-line anticoagulation in patients receiving ECCO_2_R therapy? (unfractionated heparin, low-molecular-weight heparin, bivalirudin, argatroban, regional citrate anticoagulation or other [please specify])
    2. Anticoagulation with intravenous unfractionated heparin should be preferably applied to the extracorporeal circuit? (Yes/No)
    3. If unfractionated heparin is used, do you monitor (activated partial thromboplastin time (aPTT), anti-Xa activity, both aPTT and Anti-Xa other test [please specify])
    4. If you monitor activated partial thromboplastin time (aPTT), is your target:
       1. 1.0–1.5 times normal baseline (35–45 seconds)
       2. 1.5–2.0 times normal baseline (45–70 seconds)
       3. 2.0–2.5 times normal baseline (70–90 seconds)
    5. If you monitor anti-Xa activity, is your target:
       1. 0.2–0.3 UI/mL
       2. 0.3–0.5 UI/mL
       3. 0.5–0.7 UI/mL
    6. Do you perform an initial bolus of heparin? (Yes/No, If YES, what dose?)
    7. Do you perform a bolus in patients already on full anticoagulation? (Yes/No)
    8. Do you perform a bolus:
       1. Before catheter insertion
       2. When guidewires have been inserted
       3. After catheter insertion
    9. In patients with proven HIT-2, do you follow the recommended Argatroban protocol, i.e. 0.5–2.0 µg/kg/min? (Yes/No, If no, which drug do you use?)
